# Human Granulosa Cells—Stemness Properties, Molecular Cross-Talk and Follicular Angiogenesis

**DOI:** 10.3390/cells10061396

**Published:** 2021-06-05

**Authors:** Claudia Dompe, Magdalena Kulus, Katarzyna Stefańska, Wiesława Kranc, Błażej Chermuła, Rut Bryl, Wojciech Pieńkowski, Mariusz J. Nawrocki, James N. Petitte, Bogusława Stelmach, Paul Mozdziak, Michal Jeseta, Leszek Pawelczyk, Jędrzej M. Jaśkowski, Hanna Piotrowska-Kempisty, Robert Z. Spaczyński, Michał Nowicki, Bartosz Kempisty

**Affiliations:** 1 The School of Medicine, Medical Sciences and Nutrition, University of Aberdeen, Aberdeen AB25 2ZD, UK; u16cd16@abdn.ac.uk; 2 Department of Veterinary Surgery, Institute of Veterinary Medicine, Nicolaus Copernicus University in Torun, 87-100 Torun, Poland; magdalena.kulus@umk.pl; 3 Department of Histology and Embryology, Poznan University of Medical Sciences, 60-781 Poznan, Poland; k.stefanska94@o2.pl (K.S.); mnowicki@ump.edu.pl (M.N.); 4 Department of Anatomy, Poznan University of Medical Sciences, 60-781 Poznan, Poland; wkranc@ump.edu.pl (W.K.); rutbryl@gmail.com (R.B.); mjnawrocki@ump.edu.pl (M.J.N.); 5 Division of Infertility and Reproductive Endocrinology, Department of Gynecology, Obstetrics and Gynecological Oncology, Poznan University of Medical Sciences, 60-535 Poznan, Poland; blazej.chermula@wp.pl (B.C.); b.stelmach@wp.pl (B.S.); pawelczyk.leszek@ump.edu.pl (L.P.); rspaczynski@yahoo.com (R.Z.S.); 6 Division of Perinatology and Women’s Diseases, Poznan University of Medical Sciences, 60-535 Poznan, Poland; wpienkowski@poczta.onet.pl; 7 Prestage Department of Poultry Science, North Carolina State University, Raleigh, NC 27607, USA; jnppo@ncsu.edu (J.N.P.); pemozdzi@ncsu.edu (P.M.); 8 Physiology Graduate Program, North Carolina State University, Raleigh, NC 27695, USA; 9 Department of Obstetrics and Gynecology, Faculty of Medicine, Masaryk University and University Hospital Brno, 602 00 Brno, Czech Republic; jeseta.michal@fnbrno.cz; 10 Department of Veterinary Sciences, Czech University of Life Sciences in Prague, 165 00 Prague, Czech Republic; 11 Department of Diagnostics and Clinical Sciences, Institute of Veterinary Medicine, Nicolaus Copernicus University in Torun, 87-100 Torun, Poland; jmjaskowski@umk.pl; 12 Department of Toxicology, Poznan University of Medical Sciences, 60-631 Poznan, Poland; hpiotrow@ump.edu.pl; 13 Department of Basic and Preclinical Sciences, Institute of Veterinary Medicine, Nicolaus Copernicus University in Torun, 87-100 Torun, Poland

**Keywords:** stem cells, granulosa cells, cumulus cells, translational medicine, miRNA, follicular angiogenesis

## Abstract

The ovarian follicle is the basic functional unit of the ovary, comprising theca cells and granulosa cells (GCs). Two different types of GCs, mural GCs and cumulus cells (CCs), serve different functions during folliculogenesis. Mural GCs produce oestrogen during the follicular phase and progesterone after ovulation, while CCs surround the oocyte tightly and form the cumulus oophurus and corona radiata inner cell layer. CCs are also engaged in bi-directional metabolite exchange with the oocyte, as they form gap-junctions, which are crucial for both the oocyte’s proper maturation and GC proliferation. However, the function of both GCs and CCs is dependent on proper follicular angiogenesis. Aside from participating in complex molecular interplay with the oocyte, the ovarian follicular cells exhibit stem-like properties, characteristic of mesenchymal stem cells (MSCs). Both GCs and CCs remain under the influence of various miRNAs, and some of them may contribute to polycystic ovary syndrome (PCOS) or premature ovarian insufficiency (POI) occurrence. Considering increasing female fertility problems worldwide, it is of interest to develop new strategies enhancing assisted reproductive techniques. Therefore, it is important to carefully consider GCs as ovarian stem cells in terms of the cellular features and molecular pathways involved in their development and interactions as well as outline their possible application in translational medicine.

## 1. Introduction

Female infertility is a worldwide problem, affecting millions of women. It can be caused by various factors and reproductive system disorders, especially those affecting the ovaries. For example, polycystic ovary syndrome (PCOS) affects 5% to 10% of females of reproductive age [1]. The cause of PCOS is multifactorial, including both genetic and environmental factors. PCOS signs and symptoms include enlarged ovaries with numerous small cysts, irregular menstrual cycles, hirsutism, increased androgen levels and problems with ovulation [1]. Another relatively common disease affecting female fertility is premature ovarian insufficiency (POI), occurring in about 1% of all women worldwide. POI is characterised by amenorrhea, hypergonadotropism and hypoestrogenism before the age of 40, effectively causing female infertility [2]. Furthermore, ovarian cysts also constitute a serious problem, affecting both human and animal reproduction [3]. As therapies for reproductive diseases are often characterised by low efficacy, there is a need to develop new treatment strategies for possibly enhancing or restoring fertility.

Stem cells have gained a lot of interest in recent years, especially in the context of regenerative medicine and cellular therapies. As embryonic stem cell (ESC) use is associated with significant ethical concerns, stem cells isolated from adult tissues such as bone marrow [4], adipose tissue [5] or the umbilical cord [6] are an excellent alternative for cell-based therapies. This group includes “mesenchymal stem cells” (MSCs), characterised by a specific set of criteria established by the International Society for Cellular Therapy [7]. MSCs were primarily isolated from the bone marrow by Friedenstein et al. [8], but their populations have since been found in other adult tissues.

The cells building the follicle, a primary functional unit of the ovary, such as granulosa cells (GCs), were demonstrated to possess certain stem-like properties. Furthermore, GCs express markers specific for MSCs such as CD105, CD90 and CD44 and differentiate into other cell types such as osteoblasts, neurons and chondrocytes [9]. Therefore, it seems that there is a potential for their application in translational medicine. Hence, it is important to consider ovarian stem cell cellular features and the molecular pathways involved in their development and interactions as well as outline their possible applications in translational medicine. For this reasons, this article reviews the cellular and molecular aspects of human folliculogenesis as well as follicular angiogenesis, a crucial event for achieving dominance by the follicle; the stemness properties of GCs and their molecular cross-talk; and the influence of miRNAs on function of GCs and cumulus cells (CCs). Finally, we review studies concerning the potential clinical utilisation of ovarian stem cells or their derivatives.

## 2. Cellular and Molecular Aspects of Folliculogenesis

Ovaries are covered with a single layer of flattened or cuboid cells known as the coelomic epithelium, whereas the ovarian stroma consists mostly of fibroblast-like cells and can be subdivided into cortex and medulla, with ovarian follicles located in the cortex [10].

The ovarian follicle, the ovary’s basic functional unit, consists of an oocyte surrounded by one or several layers of somatic cells, including GCs and theca cells. Depending on the folliculogenesis stage, follicles’ exact appearance varies in terms of the number of somatic cell layers and their cellular structure.

Folliculogenesis begins during the fourth month of foetal life, resulting in the formation of the stock of resting follicles (RF), comprising mostly primordial follicles [11]. The primordial follicle contains an oocyte arrested in prophase I, surrounded by a flat layer of GCs enveloped by a basement membrane [12]. Subsequently, due to an unknown selection mechanism, primordial follicles undergo growth and differentiation, becoming primary follicles characterised by cuboidalisation of GCs [13]. Simultaneously, a glycoprotein layer (zona pellucida), connecting GCs with the oocyte, is formed [14].

Primary follicles develop into secondary follicles when GCs proliferate and form three to six layers of cells. Simultaneously, theca cells are recruited from connective tissue to surround the basal lamina and form an outer layer of the follicle [15]. Later, theca cells differentiate into theca externa and theca interna, the former composed of cells similar to undifferentiated theca cells and the latter resembling epithelioid cells [11]. Subsequently, these small, fluid-filled cavities develop within the follicle, resulting in antral follicle formation. The antrum enlarges in response to FSH stimulation, and the GCs surrounding the oocyte differentiate into CCs, building the cumulus oophorus, which is characteristic for the antral (preovulatory) follicle [12]. Therefore, the preovulatory follicle contain four different layers of GCs, namely the granulosa membrane on the furthest layer, which becomes vascularised with capillaries sprouting from the theca interna [16]; the periantral granulosa layer; the cumulus oophorus in the intermediate layer; and the corona radiata in the layer closest to the oocyte. GCs exert different functions in each layer, secreting various molecules and expressing a range of receptors [17,18].

Due to the LH surge at the final stage of follicular development, ovulation occurs, and GCs differentiate into luteal cells. The follicle ruptures, forming a gap filled by GCs, which transform into lutein cells with the primary purpose of maintaining the pregnancy [19]. Simultaneously, the oocyte resumes meiotic division. Furthermore, dynamic angiogenetic processes in the corpus luteum ensure a proper supply of blood containing nutrients, oxygen and hormones for the cells showing hormonal activity [20]. However, corpus luteum may regress, and production of oestradiol by lutein cells may be inhibited due to TNFα [21], which was shown to be expressed by GC during short-term in vitro culture [22].

While both CCs and mural GCs differentiate from the same common progenitor during folliculogenesis, they exert different functions [23]. CCs remain in close contact with the oocyte via gap junctions formed by trans-zonal cytoplasmic projections transverse the zona pellucida matrix, which results in cumulus–oocyte complex (COC) development [24]. Mural GCs produce oestrogen during the follicular phase and progesterone after ovulation, while CCs surround the oocyte tightly and form the cumulus oophorus and corona radiata inner cell layer. Gap junctions connecting CCs and the oocyte allow bi-directional cellular communication, exchanging nutrients and metabolites, which results in the stimulation of oocyte maturation [25,26]. Finally, both types of GCs work together toward the oocyte’s full maturation, maintaining its quality as well as conducting steroidogenesis [27]. The co-occurrence of folliculogenesis and oogenesis allows the mature Graafian follicle to protect the matured ovum [28].

Although the GCs and epithelial cells share similar characteristics, the follicular epithelium is more dynamic than other epithelia in the body, as follicular development requires significant changes. As the follicle grows, the epithelium expands and the number of layers of GCs forming the follicular epithelium grows around the oocyte, followed by their lateral expansion [29]. Furthermore, gonadotropins regulate the changes characteristic for the ovarian gland that occur from embryogenesis until menopause, while transcription factors influence gene expression during oogenesis and the development of the antral follicles. Hence, any mutations in these genes may lead to ovarian insufficiency and infertility in mammals [30].

There are several important factors involved in folliculogenesis and development of GCs. Follistatin (FST) and bone morphogenetic protein pathways are engaged in folliculogenesis, whereas the forkhead transcription factor (FOXL2) is a master regulator of GC formation [31]. The addition of growth factors such as bFGF, activin A, BMP4, wingless-type mouse mammary tumour virus integration site family member 3A (WNT3A) and follistatin to an in vitro culture of human embryonic stem cells resulted in their differentiation into granulosa-like cells [31].

## 3. Ovarian Cell Stem-Like Plasticity

Two different populations of ovarian stem cells resembling MSCs have been discovered to reside in ovarian epithelial layers. Ovarian surface epithelial cells were reported to be a possible source of germ cells, while the epithelium-derived epithelial nests might represent primitive GCs; both germ cells and epithelial nests differentiate de novo from mesenchymal progenitor cells located in the ovarian tunica albuginea. It was suggested that germ cells derived from ovarian surface epithelial cells could assemble within primitive GC nests, forming primary follicles [16]. Similarly, Bowen et al. [32] revealed that human ovarian surface epithelium was multipotential because it expressed genes associated with adult stem cell maintenance. In addition, ovarian epithelial cells in patients with severe ovarian infertility were shown to express markers of pluripotency such as SOX-2 or SSEA-4 [33].

Bhartiya et al. [34] established that the ovary contains two stem cell populations: very small, embryonic-like stem cells (VSELs) and ovarian stem cells (OSCs), both located in the ovarian surface epithelium (OSE). Pluripotent VSELs are small (2–4 µm), quiescent stem cells equivalent to primordial germ cells and are able to self-renew and give rise to OSCs, which subsequently form germ cell nests by clonal expansion. While OSCs express OCT-4A, VSELs are characterised by the expression of OCT-4B [34]. The variety in the expression of OCT-4 occurs due to asymmetric cell division of VSELs embedded in the OSE, causing relocation of OCT-4 from the nuclear membrane to the cytoplasm in MSCs [35]. In addition, VSELs present in all adult organs are thought to migrate to different tissues to replace potentially damaged cells [36]. Furthermore, both VSELs and OSCs respond to FSH, participating in the formation of oocytes and primordial follicles, which is also preceded by an epithelial–mesenchymal transition [35].

Recently, a single-cell RNA sequencing of the ovarian cortex revealed six populations of cells: oocytes, GCs, immune cells, endothelial cells, perivascular cells and stromal cells [37]. However, cortical GCs clustered separately from GCs found in antral follicles. The aim of this study was to obtain OSCs by DDX4 (DEAD-Box Helicase 4) antibody, a common marker for germ cells, isolation; moreover, OSCs were suggested to express it on their cellular membrane. The OSCs markers, such as DDX4, were found only in oocytes and perivascular cells, indicating that germline stem cells were absent in adult ovaries [37]. This would contradict the findings of Bhartiya et al. [34]; however, it is important to note that the study by Wanger et al. [37] focused on the ovarian cortex, whereas Bhartiya et al. [34] reported the presence of OSCs in OSE. Moreover, the latter study addressed the controversy regarding the utilisation of the DDX4 marker to obtain OSCs and concluded that additional markers should be used, but technical confusion should not be the reason to doubt the existence of OSCs [34]. Wagner et al. [37], on the other hand, concluded that the DDX4 antibody used in their experiment recognised an epitope specifically expressed on perivascular cells, even though they did not express the *DDX4* transcript. Because DDX4 has been found in perivascular cells, it is interesting to consider that these cells may give rise to multipotent progenitors. It would be consistent with the results obtained by Crisan et al. [38], who obtained perivascular cells from various tissues such as pancreas, muscle, adipose tissue and others. When cultured in vitro, these cells were positive for markers characteristic for MSCs such as CD44, CD73, CD90 and CD105 and exhibited trilineage differentiation potential, suggesting that perivascular cells might have given rise to stem cells, especially MSCs [38].

Another single-cell RNA sequencing study of the inner cortex of adult ovaries was recently performed [39]. As a result, different populations of GCs were distinguished based on specific gene expression. The GCs of small antral follicles exhibited high expression of WT1 (Wilms tumour 1) and EGR4 (early growth response 4) and low expression of VCAN (versican) and FST (follistatin). GCs from selectable follicles were categorised as CCs and mural GCs. CCs showed high expression of VCAN, FST, IGFBP2 (insulin-like growth factor binding protein 2), HTRA1 (high-temperature requirement A serine peptidase 1), INHBB (inhibin subunit beta B) and IHH (Indian hedgehog signalling molecule). Mural GCs expressed high levels of KRT18 (keratin 18), CITED2 (CBP/p300-interacting transactivator 2) and AKIRIN1 and low levels of WT1 and EGR4. In contrast, GCs from atretic follicles did not express VCAN, FST or KRT18 and expressed lower levels of GJA1 (gap junction protein alpha 1) and CDH2 (cadherin 2) compared to other clusters of GCs [39]. However, other cells with stem-like properties were found to reside in the ovary.

**Figure 1:**
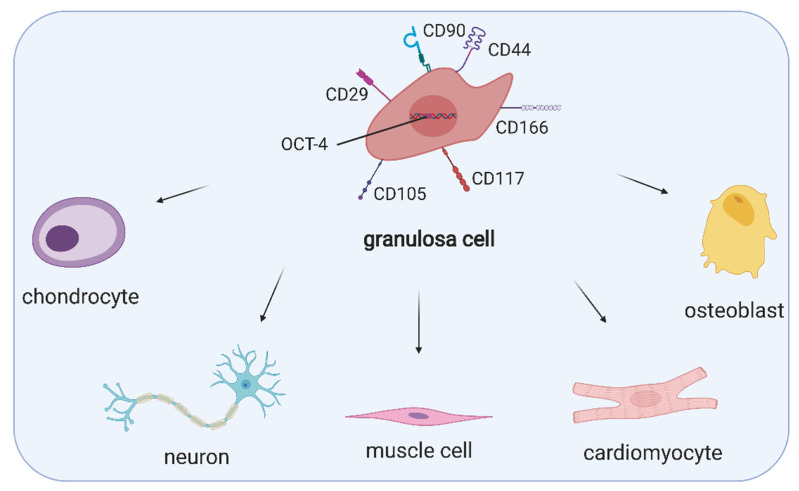
Expression of MSC- and stemness-specific markers, namely CD90, CD44, CD166, CD117, CD105, CD29 and OCT-4 as well as the multipotency of granulosa cells. (Created with BioRender.com, accession date: 29 May 2021).

As GCs proliferate rapidly during folliculogenesis and exert a variety of specialised functions, it has been suggested that their population contains cells at various stages of differentiation and therefore is not uniform. Kossowska-Tomaszczuk et al. [9] demonstrated, for the first time, that GCs had high proliferation capability and differentiation potential toward various cell lineages and were not terminally differentiated as it was previously assumed. The authors aspirated follicular fluid during oocyte collection for assisted reproduction and isolated a subpopulation of cells expressing FSHR, which were subsequently pooled from different patients. Since these cells expressed both FSHR and aromatase, they were recognised as luteinising GCs. When cultured with LIF (leukaemia inhibitory factor), commonly used to support stem cell growth, they remained viable up to 4 months and retained their morphology. Therefore, they could be cultivated in vitro for a prolonged period. Importantly, LIF is present in follicular fluid, and it has been suggested that it promotes the primordial-to-primary follicle transition [40]. These GCs were positive for OCT-4, a transcription factor considered one of the primary regulators of differentiation and self-renewal. However, other markers of pluripotency such as Nanog, Stellar and or Vasa were negative. Additionally, GCs expressed markers characteristic for MSCs such as CD166, CD90, CD105, CD29 or CD44, but not CD73. GCs were also subjected to neurogenic, osteogenic and chondrogenic differentiation, which resulted in the expression of specific markers such as nestin, neurofilament, BSP (bone sialoprotein), OC (osteocalcin) or COLL1 (collagen 1) [9]. In addition, a subpopulation of GCs was reported to express CD117 (c-kit), a mesenchymal lineage marker and stem cell factor receptor, which is assumed to be involved in the survival of human ovarian follicles [16]. Markers characteristic for stem cells, expressed by GCs, are presented in Figure 1.

Further studies showed the ability of GCs to differentiate into muscle cells and cardiac cells [41,42]. However, to maintain stemness properties, these cells may express FSHR but not LHR. It was observed that an increase in LHR expression promoted GC phenotype, and cells expressing both FSHR and LHR would enter apoptosis as in vitro culture progressed [16]. Furthermore, GCs undergo cell division in contact with neighbouring cells in the absence of the substratum that is characteristic for stem cells as well as form basal lamina in vitro, proving that this structure is usually composed of GCs [43].

Dzafic et al. [44] isolated follicular cells aspirated during in vitro fertilisation procedures and reported that these cells, consisting mostly of GCs and theca cells, exhibited expression of MSC-related genes that varied as compared to those expressed by bone marrow MSCs. *IL10, CD45, RUNX2, CD106, OCT4* and 11 other genes were significantly upregulated in follicular cells, suggesting MSC phenotypes. However, *CD73, CD90* and *CD105* were downregulated. Importantly, follicular cells were shown to express a degree of plasticity because they were successfully differentiated toward osteogenic, adipogenic and pancreatic-like cells [44].

Furthermore, GCs share some characteristics with epithelial origin cells such as the secretion of the basal lamina and the presence of adherens junctions, believed to be fundamental for the initiation of follicular growth. However, they lack desmosomes and do not express epithelial cell markers [45]. Therefore, although GCs exhibit some epithelial characteristics, they also resemble the MSC phenotype, expressing vimentin, while their luteinised counterparts express OCT-4, an essential protein involved in cell differentiation and self-renewal.

Further stemness of GCs becomes apparent at the start of ovulation, when the basal lamina is ruptured, and the inner layer of GCs becomes more loosely packed, less polarised and increasingly proliferative [45]. Another example of epithelial–mesenchymal transition in adult tissue, a rare event happening in physiology, occurs when mural GCs increase their size, forming luteinised cells [46]. Furthermore, GCs shift from steroid production of oestradiol to progesterone before the follicle ruptures and after ovulation, if they stay in contact with endothelial cells and produce an extracellular matrix, GCs undergo hypertrophy, differentiating into large luteal cells. Simultaneously, the process of centripetal angiogenesis starts from the vascular network around the follicle, the follicular basement membrane is destroyed and endothelial cells migrate toward the inner GC layer.

A blood platelet lysate was reported to stimulate luteinisation of porcine GCs by converting oestradiol synthesis to progesterone. Furthermore, platelets containing hemoattractive substances were observed to induce angiogenesis [47]. To support the idea of using platelets in ovarian regenerative medicine, it was also suggested that blood platelets regulated endothelial cell migration and GCs’ luteinisation during corpus luteum formation in human ovaries [48].

Apart from the characteristics above suggesting the stem-like potential of GCs, they also exhibit higher telomerase activity than other adult cells without stem-like properties [49]. However, telomerase activity decreases with age and is suggested to be related to primordial follicle depletion [50].

Ovarian follicular cells exhibit stem-like potential and present phenotype changes and multipotency during long-term in vitro cultures. Under specific culture conditions eventually leading to successful differentiation into multiple cell types, these stem cells can lose GC functional markers including FSHR and aromatase, and express markers characteristic of mesenchymal stem cell phenotypes [51]. Although the expression of stem cell markers in GCs including OCT-4, Nanog and SOX-2 have been described, their expression varies between different species and maturational stages of GCs [52].

Understanding the processes underlying the differentiation of GCs toward different cell lineages and the related molecular pathways of this mechanism is fundamental to uncovering other possible stemness markers of GCs, allowing for taking full advantage of their MSC-like characteristics. Improved cell cultures to prolong the lifespan of GCs are being developed. Utilising 3D cultures, employing MEF (mouse embryonic fibroblast) medium and the addition of follicular fluid and LIF were observed to prolong the lifespan, encourage proliferation and maintain cells in an undifferentiated state in vitro [9,53,54].

## 4. Regulation of Angiogenesis in Ovarian Follicles

The proper function of ovaries and ovarian follicles is maintained by continual angiogenesis, a process of new blood vessel formation from those already existing. In response to an angiogenic stimulus such as hypoxia or wounding, the endothelial and mural cells become destabilised. Subsequently, they migrate towards the angiogenic stimuli and proliferate, forming a new vessel [53].

Follicular vasculature begins to develop at the secondary follicle stage, as previously mentioned, within the theca cell layer, with the GC layer remaining avascular, separated by the basement membrane. However, GCs seem to play an important role in this process, producing angiogenic factors and influencing events occurring in the theca layer [45]. Yang et al. [54] suggested that vascular endothelial growth factor (VEGF) was a factor responsible for initial vasculature recruitment, probably due to oocyte-secreted factors influencing its expression in theca and GCs [55]. Follicular vasculature seems to be crucial in achieving dominance by the follicle, as indicated by another study conducted in bovine follicles [56], because the vascularity was shown to be positively correlated with the expression of VEGF in oestrogen-active follicles.

The gonadotropin surge occurring in the middle of the menstrual cycle results in the breakdown of the basement membrane, invasion of the blood vessels to the granulosa layer and subsequent corpus luteum formation. Therefore, luteal angiogenesis originates from the developing follicle, especially because accumulation of pro-angiogenic growth factors occurs during preovulatory follicle development [57]. Angiogenesis occurring during corpus luteum development is more intense than follicular angiogenesis because up to 85% of the cells proliferating in the corpus luteum are of vascular origin [58]. In a previous study, it was suggested that granulosa cells might participate in the vascularisation of the corpus luteum, as they were observed to express phenotypic (Tie, Tek, cKit, Flt-1, CD-31, vWF proteins) and functional (rapid AcLDL uptake and tube-forming ability in vitro) markers associated with endothelial or endothelial-like cells [59]. Furthermore, Merkwitz et al. identified the presence of somatic, KIT-positive progenitor cells in GC cultures as well as expression of CD14, CD45, CD133 or VEGF-R2. In this study, from KIT-positive cell cultures, they obtained cultures of granulosa cells or endothelial cells, showing heterogeneity of microvascular sources. These results suggested that progenitor cells could be obtained from harvested GCs and could be important for further research on the angiogenesis of the corpus luteum [60].

Amongst pro-angiogenic factors, VEGF, basic fibroblast growth factor (FGF2), platelet-derived growth factor (PDGF) and angiopoietin (ANGPT) can be distinguished, which, in general, promote the proliferation and migration of endothelial cells, whereas thrombospondin and angiostatin are factors acting in an anti-angiogenic manner, inhibiting endothelial cell proliferation and migration or stimulating their apoptosis. Although thrombospondins (both THBS1 and THBS2) were associated with decreased vascularity and proliferation of GCs in growing follicles, a study conducted in macaques revealed that the levels of THBS1 mRNA and protein were increased in the GCs of preovulatory follicles after the gonadotropin surge. In addition, the treatment of monkey ovarian microvascular endothelial cells with THBS1 resulted in increased migration, proliferation and capillary sprout formation, suggesting that THBS1 acted also in a pro-angiogenic manner during ovulation and corpus luteum formation [61]. In contrast, Garside et al. [62] demonstrated that THBS1 both inhibited angiogenesis and promoted follicular atresia via direct induction of apoptosis of GCs.

FGF2 is expressed in mature follicles and the corpus luteum and was the first angiogenic factor identified in the ovary [63]. As indicated by Grasselli et al. [64], FGF2 exerted an inhibitory effect on nitric oxide production in porcine GCs, which might be associated with its pro-angiogenic properties. Moreover, FGF2 seemed to be a critical factor regulating early luteinisation, possibly due to its response to the LH surge because LH was demonstrated to stimulate FGF2 production in dispersed luteal cells. Furthermore, the concentration of FGF2 in bovine preovulatory follicular fluid was higher in animals experiencing an LH surge [65]. The assumption of FGF2 being vital for luteal angiogenesis is consistent with the findings of Yamashita et al. [66] in cows. Intraluteal injections of FGF2 antibody resulted in decreased progesterone secretion and corpus luteum volume. Similar results were obtained for the VEGF antibody, suggesting a prominent role of both factors in corpus luteum formation and function [66].

The induction of angiogenesis is also dependent on the occurrence of hypoxia, with hypoxia-inducible factor 1 (HIF-1) being responsible for the response to oxygen deprivation. A study conducted by Calvani et al. [67] revealed that human umbilical endothelial vein cells (HUVECs) cultured under hypoxic conditions formed tube-like structures. Neutralisation of FGF2 resulted in blocked survival and sprouting of HUVECs under hypoxic conditions, whereas HIF-1A activity was required for hypoxic induction of FGF2 mRNA and protein expression [67]. Hypoxia was also reported to influence the level of VEGF, as indicated by Nishimura et al. [68], because oxygen deprivation increased the amount of VEGF mRNA and protein in cultured bovine luteal cells.

There is no doubt that VEGF is a master regulator of both follicular and luteal angiogenesis as well as new capillary formation within the ovulatory follicle because its inhibition results in a marked decrease in endothelial and granulosa cell proliferation in developing antral follicles, alongside inhibition of follicular growth and ovulation [69]. VEGFA (vascular endothelial growth factor A) exists in several isoforms as a result of an alternative splicing, with VEGFA_165_ being the most predominant protein isoform in humans [57]. It has been suggested that various isoforms of VEGFA may be differentially expressed during folliculogenesis, as indicated by a study conducted in cows. The authors investigated the effects of progesterone on the gene expression of hypoxia-inducible factor 1 alpha (HIF-1A), VEGFA_120_ and VEGFA_164_. Progesterone might have stimulated the expression of VEGFA_120_ via HIF-1A, whereas the expression of VEGFA_164_ expression was inhibited by this hormone [70]. VEGFA binds to the receptors VEGFR1 and VEGFR2, which are located on the surfaces of endothelial cells [57]. However, several co-receptors (such as neuropilin 1 and neuropilin 2) were reported to influence the effects exerted by VEGFA. Shimizu et al. [71] revealed that the neuropilin 1 (NRP-1) gene was expressed in the GCs and theca cells of pre- and post-selection bovine follicles, whereas the NRP-2 gene was expressed only in theca cells of these follicles. Moreover, the NRP-1 gene was shown to be regulated by sex hormones; namely, oestradiol increased its expression in cultured GCs, while progesterone decreased NRP-1 expression [71].

However, other factors were also reported to play important roles in follicular angiogenesis. VEGF combined with PDGF was shown to suppress angiogenesis, suggesting an antagonistic relationship between both aforementioned factors [72]. PDGF is responsible for the recruitment of pericytes, which are important constituents of microvessels, to the blood vessel wall as well as their interactions with endothelial cells [57]. PDGF acts through PDGF receptor β (PDGFRβ). Inhibition of this signalling resulted in severe haemorrhage in mice due to the blockade of pericyte recruitment in the angiogenic corpus luteum, highlighting the importance of PDGF signalling during ovarian angiogenesis [73]. In addition, intraovarian injection of an inhibitor of PDGF receptor activity caused a significant decrease in corpora lutea in rats [74].

**Figure 2:**
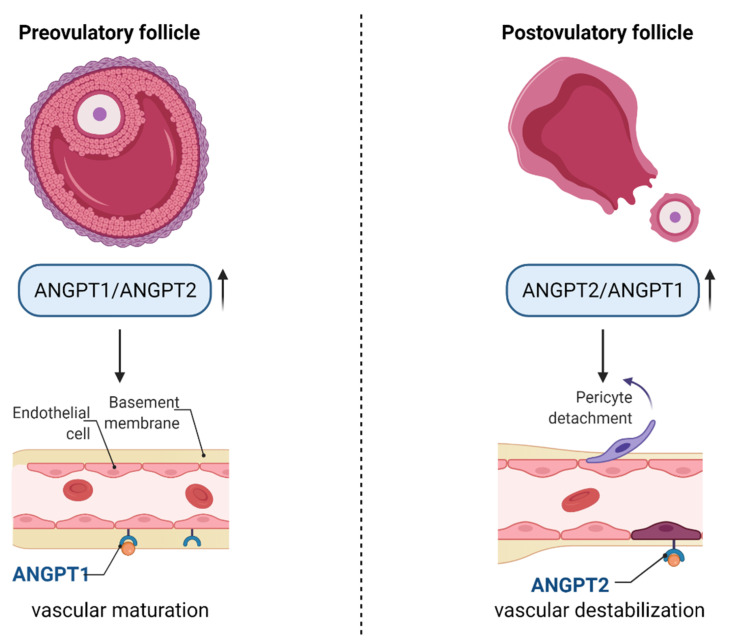
The role of angiopoietins in pre- and post-ovulatory follicles. Before ovulation, the ANGPT1/ANGPT2 ratio increases, leading to vascular maturation, whereas after the LH surge, the level of ANGPT2 increases compared to ANGPT1, leading to the destabilisation of vessels and pericyte detachment. Abbreviations—ANGPT1: angiopoietin 1; ANGPT2: angiopoietin 2. (Created with BioRender.com, accession date: 29 May 2021).

Angiopoietins (ANGPT1 and ANGPT2) act via endothelial, cell-specific tyrosine kinase receptor Tie2. ANGPT1 stimulates vessel maturation and is essential for normal vasculature development, whereas ANGPT2 is a naturally occurring antagonist for ANGPT1 and Tie2, destabilising endothelial–pericyte contacts [75]. While ANGPT1 is expressed in all tissues, ANGPT2 is present mostly in the ovaries, uterus and placenta [63]. During the pre-ovulatory stage, the ANGPT1/ANGPT2 ratio increases, suggesting that the process of vascular maturation is more evident than vascular expansion. After the LH surge, the ANGPT2/ANGPT1 ratio is increased, probably inducing destabilisation of existing vessels and pericyte detachment. In the corpus luteum, new vessels are formed due to the activity of ANGPT1 and the recruitment of pericytes, resulting in fully vascularised luteal cells [53]. Therefore, the ratio of ANGPT1/ANGPT2 changes during follicular–luteal transition and plays an important role in vessel fate, which is schematically presented in Figure 2.

## 5. The Role of miRNA in the Regulation of Granulosa and Cumulus Cells’ Function

MiRNAs are small, non-coding molecules involved in post-transcriptional gene expression regulation by base-pairing to mRNAs. They regulate various cellular processes such as proliferation, differentiation and apoptosis, targeting multiple functionally related genes constituting gene expression networks [76]. MiRNAs are expressed in ovarian tissue, GCs, oocytes and follicular fluid, among others, affecting mammalian fertility. It seems that miRNAs exert vital roles in folliculogenesis because differential expression of miRNAs involved in follicular cell proliferation, steroidogenesis, luteinisation and oocyte maturation between small and large bovine follicles, or between healthy and atretic ones, has been observed [77].

MiRNAs present in GCs or CCs may affect oocyte maturation, as indicated in several studies. Sinha et al. [78] increased or inhibited the expression of miR-130b in bovine GCs and CCs cultured in vitro, revealing that the SMAD5 and MSK1 genes were direct targets of miR-130b. Furthermore, overexpression of miR-130b resulted in increased GC and CC proliferation, whereas its inhibition during oocyte in vitro maturation caused reduction in the first polar body extrusion, mitochondrial activity and number of oocytes reaching metaphase II. Therefore, miR-130b was demonstrated to be involved in both GCs’ and CCs’ proliferation and survival and in oocyte maturation [78]. Similarly, miR-375 was reported to be involved in the regulation of bovine oocyte in vitro maturation via targeting ADAMTS1 and PGR in CCs, leading to the suppression of cumulus–oocyte complex maturation [79]. Moreover, miR-375 was shown to regulate the expression of BMPR2, thereby affecting the expression of BMP15 and GDF9 receptors, influencing the proliferation and apoptosis of bovine CCs [80]. MiR-21-3p, on the other hand, influences bovine GCs autophagy, as demonstrated by Ma et al. [81], by targeting VEGFA and attenuating PI3K/AKT signalling, resulting in autophagy inhibition.

In the case of humans, miRNAs were reported to regulate the cumulus–oocyte complex as indicated by Assou et al. [82], who compared the expression of miRNA in human metaphase II oocytes and in CCs. As a result, the most abundant miRNAs in CCs were let-7b, let-7c and miR-21, whereas miR-184 and miR-10a were the most abundant in oocytes. Further analyses revealed differentially expressed genes in CCs and oocytes that were predicted to be targeted by the aforementioned miRNAs and were associated with regulation of the cell cycle or apoptosis [82].

GCs and CCs may differ in terms of various miRNA content, as indicated by Andrei et al. [83]. Human GCs and CCs were obtained from healthy women undergoing in vitro fertilisation and subjected to small RNA sequencing. As a result, 53 miRNAs were reported to be significantly differentially expressed between GCs and CCs. Most of the highly abundant miRNAs such as miR-21-5p, let-7a-5p and let-7f-5p were present both in GCs and CCs; however, miR-30a-5p was uniquely expressed in the top 10 miRNAs of GCs, whereas miR-320a was present only in the top 10 miRNAs of CCs. Differentially expressed miRNAs were implicated to be involved in the regulation of steroidogenesis, as well as in the apoptosis and proliferation of GCs; for example, miR-146a promoted apoptosis by targeting interleukin-1 receptor-associated kinase 1 (IRAK1) and tumour necrosis factor receptor-associated factor 6 (TRAF6) [83].

MiRNAs are important regulators of GC proliferation and apoptosis. Sirotkin et al. [84] demonstrated that miR-15a inhibited the proliferation of human GCs by decreasing the level of proliferating cell nuclear antigen (PCNA). In addition, miR-15a was reported to promote the release of progesterone and testosterone, but not oestradiol, when transfected in GCs in vitro [85]. MiR-143 also participates in the regulation of hormonal production, as indicated by Zhang et al. [86]. After the transfection of miR-143 inhibitor into primary cultured GCs, the production of oestradiol was significantly increased, as well as steroidogenesis-related gene expression. Further studies revealed that miR-143 negatively regulated the signalling pathway of FSH, whereas FSH decreased miR-143 expression [86].

MiRNAs could also be implicated in various pathological conditions such as premature ovarian insufficiency or polycystic ovary syndrome. Indeed, Yang et al. [87] discovered differentially expressed miRNAs in the plasmas of POI patients and normal cycling women, revealing that miR-23a and miR-27a were highly expressed in POI patients’ plasma. After the transfection of human ovarian GCs with pre-miR-23a, there was an increase in the occurrence of apoptosis, probably due to a decrease in the X-linked inhibitor of apoptosis protein (XIAP) and caspase-3 levels [87]. Further studies aimed to clarify the exact anti-apoptotic mechanism. Using luciferase reporter assay, RT-PCR and Western blotting, Nie et al. [88] discovered that SMAD5 was a target gene for both miR-23a and miR-27, and that the regulation of apoptosis in GCs occurred via the FasL-Fas pathway. Several other miRNAs may be involved in the occurrence of POI, namely the already-mentioned miR-146a. Cho et al. [89] aimed to identify new target genes for polymorphism of miR-146a (miR-146aC > G) in POI. Altered miRNA was transfected into human GCs, and further analysis revealed that miR-146aC > G led to significantly altered regulation of cyclin D2 (CCND2) and forkhead box O3 (FOXO3), genes associated with POI. Therefore, genetic variants of miR-146a may contribute to the occurrence of POI [89]. Similarly, miR938G > A polymorphisms were also identified as factors associated with POI due to altered binding to the gonadotropin-releasing hormone receptor (GnRHR) mRNA [90]. Mir-139-5p was also reported to be involved in POI occurrence, as indicated by Zhao et al. [91]. Progesterone receptor membrane component 1 (PGRMC1), crucial for GC survival, was found to be upregulated in GCs due to hyaluronic acid-mediated suppression of miR-139-5p. In POI patients, however, levels of miR-139-5p were significantly increased, presenting inverse correlation with PGRMC1 level [91].

**Figure 3:**
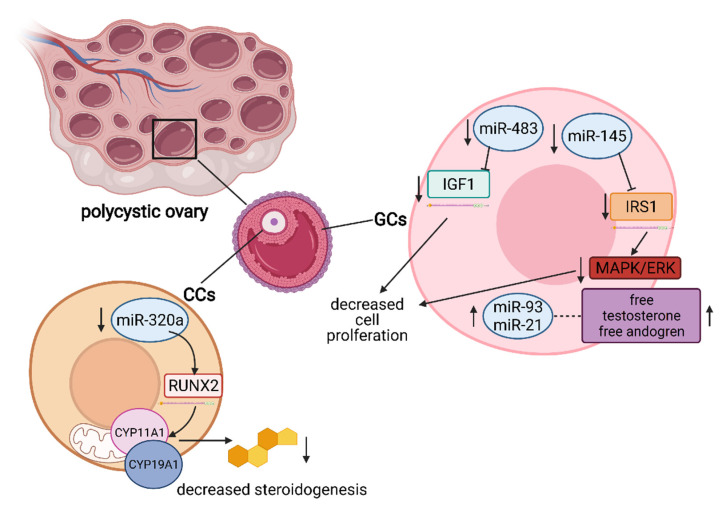
Schematic representation of miRNAs influencing the occurrence of PCOS. MiR-320a is significantly downregulated in CCs, resulting in decreased steroidogenesis due to dysregulation of RUNX2 and CYP11A1/CYP19A1. In GCs, both miR-93 and miR-21 are upregulated and positively correlated with free testosterone and the androgen index, whereas miR-483 and miR-145 are downregulated. MiR-483 directly targets the IGF1 mRNA, leading to decreased cell proliferation. IRS1 mRNA is a direct target of miR-145, leading to inhibition of the MAPK/ERK signalling pathway, resulting in decreased cell proliferation. Abbreviations—RUNX2: runt-related transcription factor 2; CYP11A1: cytochrome P450 family 11 subfamily A member 1; CYP19A1: cytochrome P450 family 19 subfamily A member 1; IGF1: insulin-like growth factor 1; IRS1: insulin receptor substrate 1; MAPK: mitogen-activated protein kinase; ERK: extracellular signal-regulated kinase; GCs: granulosa cells; CCs: cumulus cells. (Created with BioRender.com, accession date: 29 May 2021).

In the case of PCOS, miR-93 and miR-21 have been highlighted as androgen-responsive factors, positively correlated with free testosterone and the free androgen index because both aforementioned miRNAs were increased in GC-form hyperandrogenic PCOS patients as compared to normoandrogenic patients [92]. Oestrogen deficiency is a hallmark of PCOS, and downregulation of miR-320a expression in CCs from PCOS patients has been found to influence steroidogenesis due to modulation of CYP11A1 and CYP19A1 by directly targeting osteogenic transcription factor RUNX2 [93]. MiR-483 was also found to be downregulated in PCOS, and its overexpression resulted in decreased cell viability and proliferation as well as cell cycle arrest induction in human granulosa-like tumour cell line KGN. Such inhibition of proliferation was a result of miR-483-targeting IGF1 [94]. MiR-145 negatively regulates the proliferation of GCs from PCOS patients. However, miR-145 inhibited the expression of insulin receptor substrate 1 (IRS1), which resulted in inhibition of the activation of p38 mitogen-activated protein kinase (p38 MAPK) and extracellular signal-regulated kinase (ERK). Furthermore, high concentrations of insulin decreased the expression of miR-145, upregulated IRS1 and promoted cell proliferation, which was consistent with the downregulation of miR-145 in PCOS patients [95]. The important miRNAs and their functions in PCOS are presented in Figure 3.

## 6. Ovarian Follicular Cells’ Molecular “Cross-Talk” and Interaction

The oocyte’s developmental ability to undergo meiosis, be fertilised and form a healthy embryo is a factor determining female fertility. The growth of the mammalian oocyte is simultaneous with follicular growth, both of which are regulated via signals from molecular cross-talk between the germline and somatic cells. The oocyte grows within the follicle, which also comprises the somatic granulosa or theca cells, extending through an inward division of the outermost layer that results in additional layer formation [14]. When the antrum develops, GCs differentiate into CCs and mural GCs [23]. Meanwhile, the oocyte grows and develops the ability to undergo meiosis and be fertilised before ovulation, supported by GCs [96,97]. Folliculogenesis, oogenesis and ovulation are regulated by pituitary gonadotropins (reviewed by Amsterdam A et al. [98], whose action is mediated by intra-ovarian signals from paracrine factors and cell–cell communication through gap junctions [6]. Specifically, the bi-directional communication between the oocyte and CCs is crucial for proper folliculogenesis and oogenesis. CCs strongly support the growth and maturation of the oocyte, which, in turn, influences proliferation and differentiation of CCs as well as their production of extracellular matrix and steroid hormones. Moreover, both GCs and CCs protect the oocyte from oxidative stress through the antioxidant system [99,100].

Considerable evidence suggests the involvement of CCs in the development of the oocyte. However, only a few paracrine factors have been recognised. KIT ligand (KITL) and the KIT tyrosine kinase receptor mediate the CC–oocyte interaction and are both expressed in developing and preovulatory follicles of the postnatal ovary. KITL is expressed in rat, mouse and human CCs and was reported to stimulate the oocyte’s growth through the KIT receptor located on the oolemma. Moreover, it was identified as necessary and sufficient to induce primordial follicle development and might trigger oocyte’s growth initiation [101,102,103,104,105]. Mice with mutations in KIT or KITL were reported to be infertile, and KITL-dependant activation of KIT is essential for ovarian follicle growth when FSH is not yet expressed [106,107]. KITL binding to KIT activates diverse signalling pathways involved in cell survival and apoptosis. Furthermore, phosphatidylinositol (PI) 3-kinase (PI3K) is a KIT effector, leading to changes in the expression of key players of the apoptotic pathway [108]. PI3K inhibitors were shown to block the anti-apoptotic effect of KITL in germ cells during foetal oogenesis. Simultaneously, PTEN negatively interacted with the PI3K signalling pathway, and oocyte-specific PTEN knockout led to premature ovarian insufficiency [109]. Furthermore, KIT activation induced the phosphorylation of AKT and FKHRL1, activating the former and functionally suppressing the latter [110].

**Figure 4:**
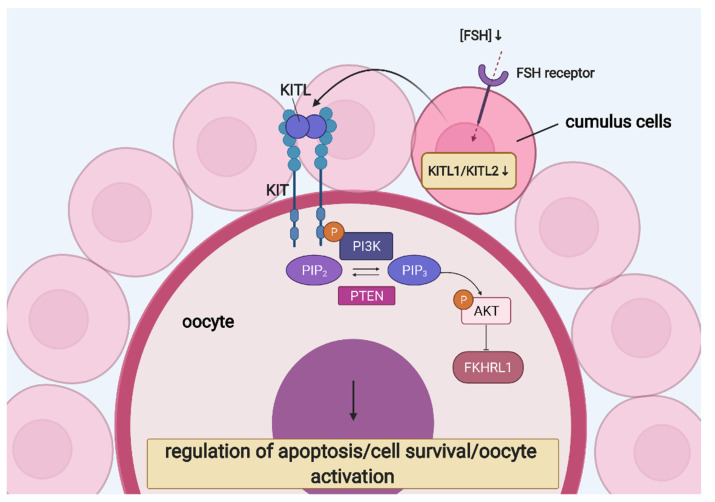
KIT signalling in CC–oocyte interaction: regulation of oocyte growth and development. Abbreviations—AKT: protein kinase B; FKHRL1: forkhead1; FSH: follicle-stimulating hormone; KITL: KIT ligand; PI3K: phosphatidylinositol 3-kinase; PIP_2_: phosphatidylinositol 4,5-bisphosphate; PIP_3_: phosphatidylinositol (3,4,5)-trisphosphate; PTEN: phosphatase and tensin homolog. (Created with BioRender.com, accession date: 29 May 2021).

In CCs, KITL is expressed as either a soluble or membrane-bound protein, which, respectively, can be cleaved or not (remaining stably on the membrane). Furthermore, the latter (KITL2) is thought to be the principal isoform necessary for the oocyte’s growth, whereas the former (KITL1) is associated with fully grown oocytes [111]. FSH is the main endocrine factor regulating ovarian functions and stimulating follicle growth. It influences the expression of KITL—its low concentration increases KITL2 level and stimulates oocyte growth, while high expression increases the KITL1/KITL2 ratio, failing to promote development of the oocyte. Therefore, the correct concentration of FSH is crucial for proper development of the oocyte [105]. The role of KIT signalling in CC–oocyte interaction is presented in Figure 4.

Furthermore, the understanding of molecular mechanisms and regulation of gene expression in CCs may shed light on oocyte quality and their ability to acquire developmental competence. Long-term in vitro cultured CCs were demonstrated to exhibit significant changes in the expression of genes such as *DKK1*, *ANXA3*, *KIAA1199*, *VCAM1* and *HTR2B*, all of which were upregulated. *DKK1* is an antagonist of the WNT signalling pathway responsible for pluripotency regulation. Therefore, it was hypothesised that elevated expression of DKK1 in CCs might influence oocyte developmental competence acquisition and sustain its pluripotency [100].

Oocyte or oocyte-secreted factors contribute to the suppression of luteinisation and are required for successful cumulus expansion and extrusion of the oocyte–cumulus cell mass from the follicle at ovulation [112]. This process depends on gonadotropins, epidermal growth factor (EGF) and paracrine factors secreted from the oocyte. CCs respond to these signals thanks to oocyte-secreted factors, leading to the expression of the transcripts necessary to form the extracellular matrix [113,114]. Oocytes stimulate proliferation of CCs and secrete paracrine factors, which inhibit LH receptors and progesterone production in CCs [115,116,117].

Oocyte-secreted factors also act in an anti-apoptotic manner, resulting in a low incidence of apoptosis in CCs. A study conducted on bovine COCs revealed that the removal of the oocyte from this complex resulted in increased CC apoptosis. This effect was reversed by the addition of denuded oocytes to CC in vitro culture, which, on the molecular level, promoted the expression of anti-apoptotic BCL-2 and inhibited pro-apoptotic BAX expression. Bone morphogenetic protein 15 (BMP-15) and bone morphogenetic protein 6 (BMP-6), both secreted by the oocyte, maintained the anti-apoptotic effect via establishment of a localised gradient of bone morphogenetic proteins [118]. During long-term in vitro culture of human CCs, the expression of the apoptosis-regulatory genes BAX, CASP9 and TP53 decreased, as indicated by the results of RT-qPCR [119]. However, the study conducted with GCs cultured in vitro for seven days revealed that the ratio of BCL-2/BAX was improved after that period, indicating these cells’ survival [120].

Furthermore, BMP-15 and growth differentiation factor 9 (GDF-9) are fundamental for follicular development [121,122]. Oocytes communicate via these factors, and their secretion is regulated by bi-directional communication between oocytes and CCs [123]. These oocyte-secreted factors prevent spontaneous luteinisation of CCs and control their endocrine function [124,125]. Oocytes produce GDF-9, deficiency in which causes an arrest of follicular development, leading to infertility. The absence of BMP-15, on the other hand, results in decreased ovulation and fertilisation rates. These two factors belong to the TGF-β superfamily and are essential for regulating the differentiation and proper functioning of CCs [126]. The superfamily of TGF-β consists of factors crucial in mammalian reproductive functions, participating in folliculogenesis, oogenesis and embryo development [127].

BMP-15 upregulates KITL1 and KITL2. However, the ratio of KITL1/KITL2 remains unchanged, which is crucial for the stimulation of oocyte development. The soluble isoform of KITL inhibits BMP-15 expression in oocytes. Therefore, a negative feedback loop between KITL and BMP-15 may be a primary regulatory pathway [105]. Moreover, because FSH influences KITL expression, and its high concentration decreases BMP-15 expression, FSH may regulate BMP-15 expression via KIT signalling [105].

GDF-9 may inhibit the expression of KITL. Similarly, KITL expression was inhibited by GC co-culture with fully grown, but not with partly grown, oocytes. Therefore, it was suggested that GDF-9 mediated the effect of fully grown oocytes on KITL expression in GCs [128].

Additionally, other components of the TGF-β superfamily were reported to play an essential part in follicular cell cross-talk. The expression of TGF-β1 after 7 days in vitro culture was unchanged, probably due to the regulatory functions of GC proliferation and differentiation as well as stimulation of preantral follicle growth [129].

CCs maintain contact with the oocyte via cellular processes known as transzonal projections (TZPs), which penetrate the zona pellucida, an extracellular coat formed around the oocyte after it enters the growth phase [130]. TZPs originate from CCs and are composed of a strong backbone of actin filaments or tubulin [131,132]. Microtubule-containing TZPs are assumed to be involved in paracrine communication, whereas actin-containing TZPs appear to mediate gap junctional communication and CC–oocyte adhesion. FSH was proven to be an essential modulator of microtubule-containing TZP organisation [24]. The amount and shape of TZPs change during follicular development, with a high number contributing to growth and a lower number during the maturation of fully grown oocytes upon gonadotropic surges [133]. Gap junctions form at the tip of these TZPs and are composed of connexins, a family of 20 proteins [134]. Six connexins form a connexon that, together with another connexon from an adjacent cell, forms a channel between cells, connecting the oocyte and CCs or two CCs in the follicle [135,136]. Amino acids are transported through these gap junctions, enhancing uptake of the glycine, alanine, lysine and taurine necessary for oocyte growth [137]. Other molecules passing via gap junctions include ions, metabolites and cAMP. Finally, mammalian oocytes rely on CCs containing additional GLUT that have a high affinity to glucose as well as phosphofructokinase activity to convert glucose into the substrates necessary for energy metabolism during the oocyte’s growth [138,139].

Apart from bi-directional communication between the oocyte and CCs or GCs, ovarian follicular cells also interact with each other. Both GCs and theca cells produce steroidal and nonsteroidal factors, influencing each other’s proliferation and differentiation during folliculogenesis. Specifically, growth factors secreted by theca cells such as epidermal growth factor (EGF), transforming growth factor α (TGFα), keratinocyte growth factor (KGF), hepatocyte growth factor (HGF) and bone morphogenetic protein 7 (BMP-7) are thought to promote the proliferation of GCs and suppress their apoptosis in early antral follicles [140]. Moreover, theca cells produce androgens, with androgen receptors localised on GCs. In mice, these theca cell-derived androgens were shown to stimulate GC mitosis and preantral follicle growth, as indicated by in vitro studies [141].

The molecular interaction between the oocyte and theca cells has been described, as well. As previously mentioned, GDF-9 belongs to the oocyte-secreted factors. A study conducted in rats revealed that GDF-9 enhanced follicular development, while its antagonist suppressed follicular growth and androgen production. Moreover, when the androgen receptor antagonist flutamide was used, it suppressed the preantral follicle growth mediated by GDF-9 in vitro. Therefore, GDF-9 was proposed to play a role in the promotion of preantral follicle growth via androgen synthesis upregulation by theca cells [142].

## 7. Application of Ovarian Follicular Stem Cells in Translational Medicine

Although ovarian follicular cells exhibit promising properties such as stem-like potential and high proliferation capability, the data on their possible application in translational medicine remain minimal. Because the ability of GCs to differentiate into osteogenic cells had already been suggested, Mattioli et al. [143] aimed to understand the osteo-regenerative potential of GCs. Mattioli et al. isolated GCs from growing and luteinising porcine follicles and subjected them to osteogenic differentiation, which resulted in marked extracellular matrix mineralisation, alkaline phosphatase activation, upregulation of osteocalcin and Runx2 expression. GCs were also differentiated after incorporating polylactic-co-glycolic acid (PLGA) scaffolds and being subsequently transplanted subcutaneously in the dorsal region of SCID mice. After the implant retrieval, the viable GCs surrounding nodules of calcification were revealed. The above results indicated that GCs possessed osteogenic potential both in vitro and in vivo and therefore could be used for bone regeneration [143]. Chandramohan et al. [144] proposed a scaffold based on chitosan and polycaprolactone (PCL) for ovarian stem cell engineering. The cells were collected from human follicular fluid and exhibited properties of MSCs while seeded on the chitosan/PCL scaffold, coated with zinc divalent ions to impart osteogenic properties. As a result, calcium deposition and alkaline phosphatase activity were increased, together with the expression of Runx2, osteonectin or osteocalcin, indicating that such a scaffold was compatible with MSCs and could be used in bone tissue engineering [144]. GCs were also demonstrated as a useful tool for obtaining functional oocytes. Tian et al. [145] isolated GCs from adult mouse ovaries and induced them to generate germline-competent, pluripotent stem cells (gPSCs) by a chemical approach, using crotonic sodium or acid. These gPSCs could then be differentiated into primordial germ cell-like cells and form functional oocytes [145].

Apart from the possibility to use the cells themselves, the supernatant of cultured granulosa–cumulus cells was proven to exhibit therapeutic properties [146]. Madkour et al. isolated immature denuded oocytes from patients with polycystic ovary syndrome, which were subsequently subjected to in vitro maturation (IVM) with the addition of autologous or heterologous follicular fluid and supernatant of cumulus–granulosa cells. The latter resulted in an improved yield of the developed blastocyst and an IVM rate higher than that of an in vivo maturation rate. Therefore, the supernatant of cumulus–granulosa cells could be a useful IVF-enhancing tool [146]. Atrabi et al. [147] investigated the influence of the conditioned medium of GCs and CCs on the activation of primordial follicles in mice. One-day-old mice ovaries were cultured for six days with conditioned media of GCs or CCs or co-cultured with these cells. Obtained results indicated that the conditioned medium of GCs could contribute to primordial follicle activation, probably due to downregulation of PTEN [147].

**Figure 5:**
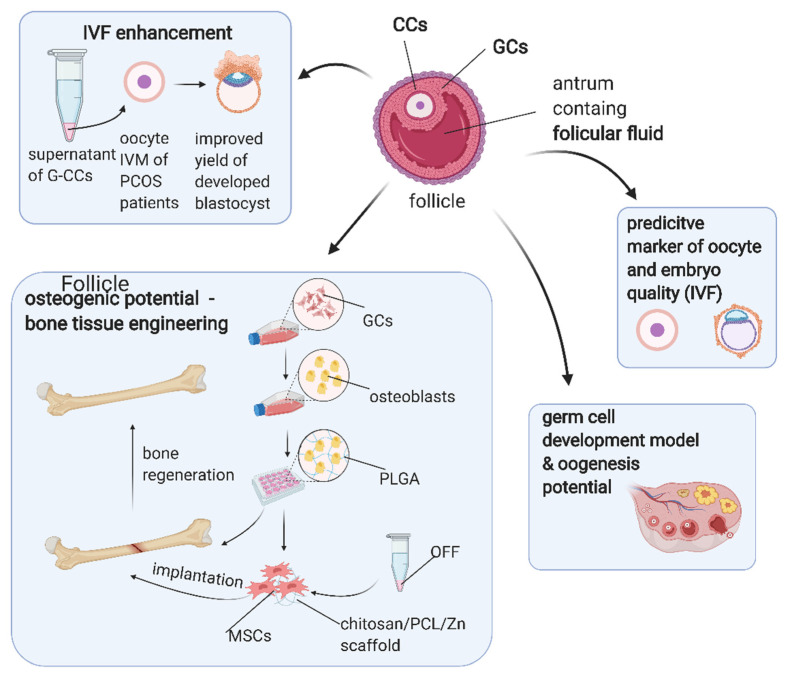
Possible clinical applications of ovarian follicular cells. Abbreviations—CCs: cumulus cells; G–CCs: granulosa–cumulus cells; GCs: granulosa cells; IVF: in vitro fertilisation; IVM: in vitro maturation; MSCs: mesenchymal stem cells; OFF: ovarian follicular fluid; PCL: polycaprolactone; PCOS: polycystic ovarian syndrome; PLGA: polylactic-co-glycolic acid. (Created with BioRender.com, accession date: 29 May 2021).

Ovarian stem cells could serve as an in vitro model to examine germ cell development because they are able to differentiate into oocyte-like cells. As indicated in a study by Taheri et al. [148], such differentiation could be induced with BMP-15. Lee et al. [149], on the other hand, overexpressed OCT-4 in ovarian stem cells, which resulted in higher oogenesis potential compared to the controls. In addition, CC and GC biomarkers may be useful in predicting oocyte and embryo quality in assisted reproductive techniques [12,150]. For example, matrix metalloproteinases (MMPs) and their tissue inhibitors (TIMPs) in GCs and CCs were assessed as potential biomarkers of oocyte quality by Luddi et al. [151]. As a result, the expression of MMP2 in GCs was shown to be increased in infertile patients as compared to fertile patients. Increased expression of MMP2 in GCs and CCs was also associated with the production of fewer oocytes, while the expression of MMP9 in GCs was positively correlated with the number of retrieved oocytes [151]. The potential application of ovarian follicular cells or their derivatives in translational medicine is presented in Figure 5.

## 8. Conclusions

Follicular cells contained within the ovary exhibit several important functions during folliculogenesis and oogenesis. They comprise cells like GCs, CCs or theca cells, involved in the synthesis, expression and metabolism of various hormones essential for gamete maturation, regulation of ovulation and pregnancy sustenance such as progesterone production upon ovulation and oestradiol production during the growth of the follicle. Moreover, they form gap junctions with the oocyte, which enable a bidirectional exchange of nutrients and metabolites, essential for gamete maturation stimulation. The developing oocyte influences follicular cell proliferation and expansion, and regulation of follicular angiogenesis is crucial for achieving dominance by the follicle. The proper function of GCs and CCs is dependent on miRNAs, and alterations in these interactions may lead to PCOS or POI. Since GCs were suggested to exhibit properties characteristic of mesenchymal stem cells such as multilineage differentiation potential, specific antigen pattern expression and high proliferation rate, their potential utilisation in translational medicine has been suggested. Although the available data are still limited, ovarian stem cells have been successfully used for bone tissue engineering, in vitro maturation and predicting the quality of oocytes and embryos. However, further studies are required to fully exploit these cells’ potential in regenerative and experimental medicine.

Following the discovery by Kossowska-Tomaszczuk et al. of the multipotency of GCs to differentiate in other cell lineages, their long-life span may be applied in a broad range of therapies and medical research specifically related to the treatment of ovarian pathologies such as cancer, endometriosis and polycystic ovary syndrome [9].

Furthermore, as prolonged cultures of GCs with LIF have shown the ability to differentiate toward different lineages both in vitro and in vivo, they exhibit characteristics of mesenchymal cells. These, in turn, have been widely studied for their therapeutic potential, with further developments potentially achieved through the study of differentiation-promoting agents. Furthermore, CGs could be employed as starting materials to obtain tissue made up of cells of other lineages, with these advances potentially representing a breakthrough for modern clinical medicine.
